# Use of a newly developed minimally invasive bilateral fixed angle locking system in the treatment of pathological pelvic fractures: a case series

**DOI:** 10.1186/s12957-024-03551-x

**Published:** 2024-10-08

**Authors:** Mark Unthan, Ivan Marintschev, Christian Spiegel, Gunther O. Hofmann, Wolfram Weschenfelder

**Affiliations:** 1 Department of Trauma, Hand and Reconstructive Surgery, Jena University Hospital, Friedrich Schiller University, Am Klinikum 1, 07747 Jena, Germany; 2 Department of Trauma, Orthopedics and Spine Surgery, Catholic Hospital “St. Johann Nepomuk”, Haarbergstr. 72, 99097 Erfurt, Germany

**Keywords:** Pathologic pelvic fracture, Sacral fracture, Minimal invasive pelvic surgery

## Abstract

**Background:**

Metastatic bone disease (MBD) and its complications have a significant impact on patients’ quality of life. Pathological fractures are a particular problem as they affect patient mobility and pose a high risk of non-union. The pelvis is frequently affected by MBD and its fixation is challenging. We present a case series of three pathological sacral fractures treated with a new minimally invasive bilateral fixed angle locking system.

**Case presentation:**

Case 1 and 2 suffered a pathological transforaminal sacral fracture without adequate trauma in stage 4 carcinomas (gastric cancer and breast cancer). Both were initially treated with non-surgical treatment, which had only a limited effect and led to imminent immobility. Both were operated on with fluoroscopic navigation and underwent transsacral SACRONAIL^®^ stabilisation according to CT morphology (S1 + S2 and S1 respectively). Immediately after the operation, pain decreased and mobilisation improved. Case 3 had a pathological transalar sacral fracture during the 2nd cycle of chemotherapy due to non-Hodgkin’s lymphoma. He soon became immobile and could only move in a wheelchair. The operation was performed with CT navigation due to the very small corridors and an implant was inserted in S1 and S2. The patient reported immediate pain relief and his ability to walk improved over the following months. Despite continued chemotherapy, no wound complications occurred.

**Conclusions:**

The cases show the advantages of the minimally invasive bilateral fixed angle locking system SACRONAIL^®^ in the treatment of patients with pathological sacral fractures. It allows immediate full weight bearing and the risk of secondary surgical complications is low. All cases showed an improvement in pain scores and mobility.

## Background

Metastatic Bone Disease (MBD), the spread of cancer cells from a primary tumour to bone, typically results in pain, pathological fractures, hypercalcaemia, spinal cord compression and other complications with significant impact on patients’ quality of life [1–4]. Due to better cancer survival rates the number of patients presenting with MBD is increasing and projections estimate a total of 2.5 million cancer patients in 2040 in the UK [5]. The most common visceral carcinomas causing orthopaedically relevant bone metastases are breast (28%), lung (17%) and renal (15%) cancers [6]. In addition, a relevant number of patients with haematological tumours (multiple myeloma and lymphoma) require surgical treatment due to the resulting osteolysis. The proximal femur is most frequently operated on, but the pelvis is also affected by relevant MBD in 16% of cases.

Pathological fractures pose a particular problem, as patients are usually already weakened and vulnerable due to the underlying disease. Additionally, pathological fractures have a high rate of non-unions and related implant failures [7, 8]. For this reason, prophylactic treatment of impending pathological fractures is more cost-effective and has a better functional outcome by preserving the patient’s independence [9, 10]. While the Mirels’ scoring system (MSS) used for long tubular bones enables a relatively good prediction of pathological fracture risk, no such scoring system has been established for the pelvis to date, which makes the treatment of patients with bone lesions in this area even more difficult [11–13]. Typical surgical options for the treatment of MBD include osteosynthesis, endoprosthesis, cryotherapy, radiofrequency ablation and cementoplasty; typical non-surgical options are radiotherapy, embolization, chemotherapy and bisphosphonates/denosumab [4].

For the surgical treatment of traumatic fractures of the posterior pelvic ring, percutaneous iliosacral screws or, in the case of more complex involvement of the sacrum or spinopelvic dissociation, spinopelvic fixation are traditionally used [14, 15]. In this context, fragility fractures of the pelvis (FFP) pose a particular problem due to poorer bone quality and associated slower bone healing, which is closer to the reality of pathological fractures. Both transsacral stabilisation and spinopelvic fixation are used for FFP, with Mendel et al. 2021 demonstrating significant outcome improvement and fracture healing. However, the subjective outcome in this study was better with transsacral stabilisation [16]. Gras et al. showed in 2015 that 88% of the population had a sufficient S1 or S2 corridor for an intraosseous transsacral implant on CT [17]. Based on this work, an angle-stable transsacral nail (SACRONAIL^®^, SIGNUS, Alzenau, Germany) was developed that can be locked in both ilia and showed no implant failure or malpositioning in the first pilot study by Marintschev et al. in a 1-year follow-up with immediate postoperative full load-bearing capacity [18].

For pathological fractures of the pelvis, various procedures have been described, particularly minimally invasive ones, which involved filling the defect with bone cement (e.g. sacroplasty) with and without screw fixation or percutaneous screw stabilisation alone, all of which were able to increase the VAS and the patient’s mobility [19, 20]. The combination of bone cement and screw fixation showed the highest biomechanical stability in the model [21]. Lee et al. have supplemented this procedure with an additional ablation of the lesion as “Ablation, Osteoplasty, Reinforcement and Internal Fixation” (AORIF) and were able to show good results in their case series [22, 23].

In our view, the disadvantage of cemented screws is their spatial limitation and thus less broad anchoring in the existing pelvic bone than a wide-span intraosseous implant such as the SACRONAIL^®^ could offer. Due to the very good results in the pilot study by Marintschev et al. in FFP, we have used the implant in pathological fractures of the posterior pelvic ring and present this case series below.

This study was approved by the local ethics committee. Patients provided written informed consent to participate in the study, which included preoperative and postoperative assessments of pain using the Numeric Rating Scale (NRS); evaluation of pain medication use; assessments of walking distance and walking time; and measurement of quality of life using the SF-12 questionnaire. Additionally, we monitored medical and implant-related complications. The structured follow-up consisted of consultations at 3 and 12 months, as well as telephone interviews at 6 and 9 months.

The inclusion criteria were skeletal involvement from an underlying oncological or haematological disease without curative resection intent, along with pathological sacral fracture and no symptom improvement following conservative therapy (outpatient for 4 weeks vs. inpatient 1 week). The exclusion criteria included ilium involvement, extensive osteolysis of the sacral body requiring spinopelvic fixation, or a poor prognosis for patients in palliative care, as determined by the multidisciplinary tumour board.

## Case presentation

### Case 1, 71 years, female, gastric cancer, adenocarcinoma, stage IV

**Fig. 1:**
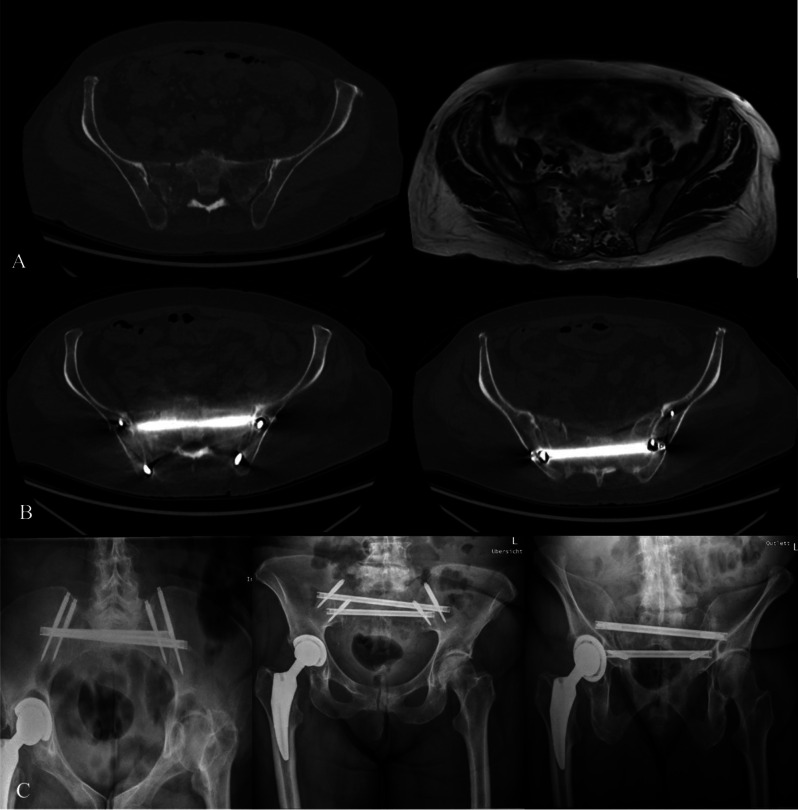
Patient 1 **A**- Axial CT and MRI scan presenting pathological sacral fracture, **B**- postoperative CT, **C**- X- ray postoperative

A 71-year-old female presented with gastric cancer, specifically adenocarcinoma, staged as IV based on UICC guidelines. The patient was referred to our clinic after 4 weeks of unsuccessful conservative management for pathological unilateral transforaminal sacral fracture due to MBD (Fig. 1). Surgical intervention was deemed necessary. The procedure involved the use of two SACRONAIL^®^ guided by a fluoroscopic navigation system (Brainlab, Munich, Germany), utilizing preoperative CT images. The surgery was successful without any associated complications. Postoperatively, the patient was advised for full weight-bearing, which she achieved with the assistance of walking aids. The patient reported a significant decrease in pain intensity, from 8/10 to 5/10 on the NRS. Both postoperative CT and X-ray modalities demonstrated correct implant positioning, without any intraforaminal penetration. Opioid use was notably reduced (Table 1), leading to the patient’s discharge after an 11-day hospital stay. Patient satisfaction, as per the NASS patient satisfaction score [24], was reported as 2, indicating an acceptable outcome despite the limited improvement in health status. Unfortunately, the patient passed away 111 days post-surgery due to the aggressive nature of the underlying tumor.

**Table 1:** Patient follow- up after minimally invasive bilateral fixed angle locking system

	Patient 1, 71years, gastric cancer stage IV (UICC)	Patient 2, 47 years, breast cancer stage IV (UICC)	Patient 3, 59 years, Non-Hodgkin lymphoma stage IVb (Ann- Arbor)
**Pre- Treatment**			
Pelvic Pain on NRS (0–10)	8	8	6
Distance of walking in meter	10	100	0
Time of Walking at once in minutes	5	20	0
Pain medication on WHO Analgesic Ladder	III daily	III daily	III daily
Quality of Life, SF 12			
PCS-12 (Physical Score)	23.4	31.3	24.3
MCS-12 (Mental Score)	17.1	34.1	34.3
Intraoperative Complications	No	No	No
**Post- Treatment Status at Hospital Discharge**			
Pelvic Pain on NRS (0–10)	5	3	0
Distance of walking in meter	100	500	0
Time of Walking in minutes	10	20	0
Pain medication on WHO Analgesic Ladder	III at demand	III daily	I
Patient satisfaction (NASS Score)	2	2	1
Quality of Life, SF 12			-
PCS-12 (Physical Score)	22.2	37,9	-
MCS-12 (Mental Score)	22.5	26,1	-
Status of Ambulation as recommended	Full weight bearing	Full weight bearing	-
Status of Ambulation	Full- weight bearing, walking aids	Full weight bearing, walking aids	-
Stay in Hospital after surgery in days	11	9	-
**3- Months follow Up**			
Pelvic Pain on NRS (0–10)	2	4	-
Distance of walking in meter	0	1000	-
Time of Walking in minutes	0	-	-
Pain medication on WHO Analgesic Ladder	III daily	III daily	-
Patient satisfaction	-	1	-
Quality of Life, SF 12	-		-
PCS-12 (Physical Score)	-	28.5	-
MCS-12 (Mental Score)	-	30.3	-
	-		
**12- Months follow Up**	-		
Pelvic Pain on NRS (0–10)	-	8	-
Distance of walking in meter	-	1000	-
Time of Walking in minutes	-		-
Pain medication on WHO Analgesic Ladder	-	Stage II daily	-
Patient satisfaction	-	2	-
Quality of Life, SF 12	-		-
PCS-12 (Physical Score)	-	27.8	-
MCS-12 (Mental Score)	-	49.8	-
Deceased	111 day post-surgery		

NASS patient satisfaction index (1- the treatment met my expectations, 2- I did not improve as much as I had hoped, but I would undergo the same treatment for the same outcome, 3- I did not improve as much as I had hoped, and I would not undergo the same treatment for the same outcome, 4- I am the same or worse than before treatment)

### Case 2, 47 years, female, invasive ductal breast cancer, stage 4

**Fig. 2:**
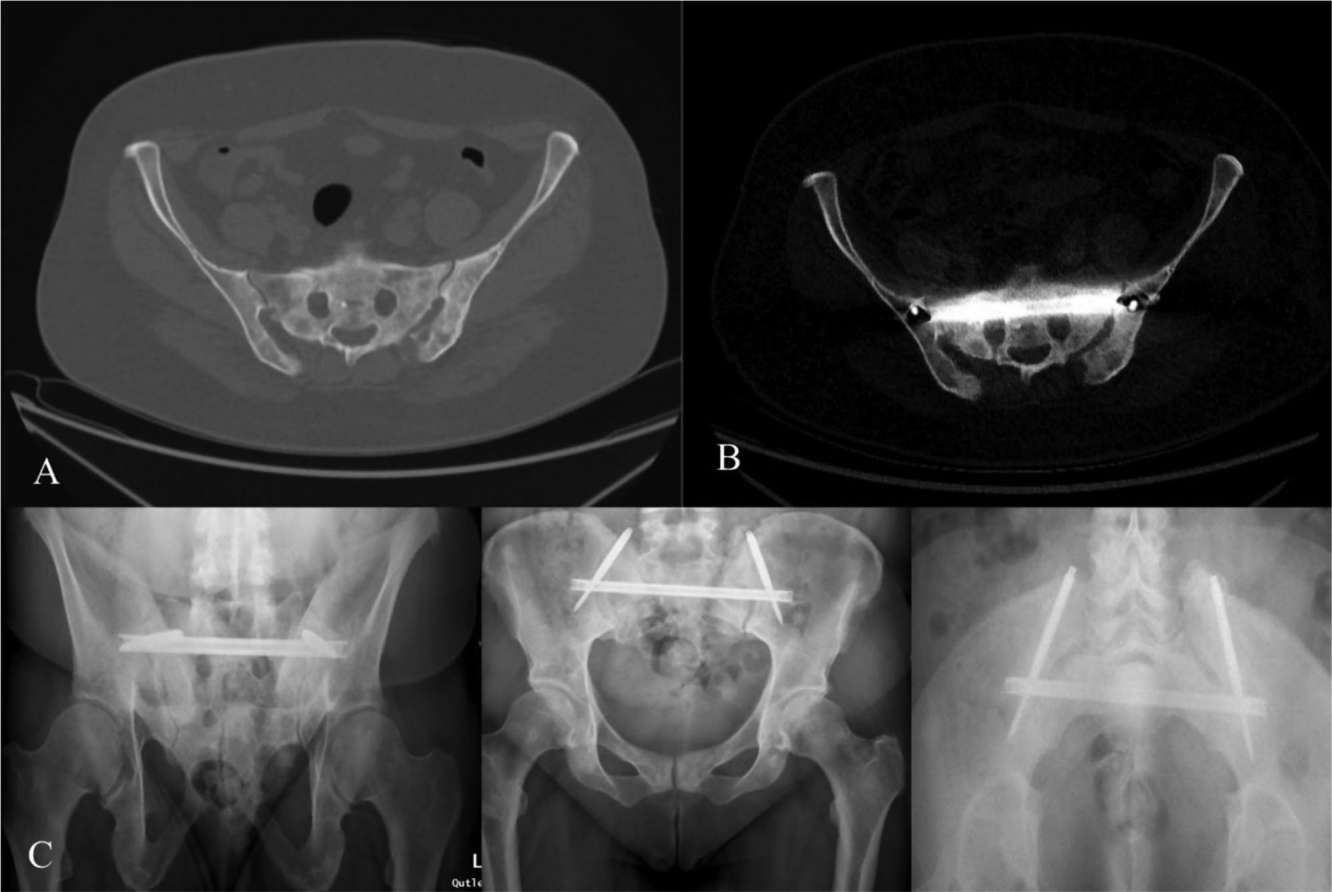
Patient 2 **A**- preoperative CT scan showing sacral fracture, **B**- postoperative CT scan, **C**- X-rays demonstrating no implant failure 12 months postoperative

A 47-year-old female with invasive ductal breast cancer in stage 4, presented with a decline in walking ability and increased pain. Imaging showed a mixed lesion involving the entire sacrum and dorsal ilium with unilateral transforaminal fissure (Fig. 2). Non-operative pain treatment showed limited efficacy. Surgical intervention involved the placement of a single SACRONAIL^®^ using fluoroscopic navigation guidance. The patient reported adequate pain relief post-surgery and self-assessment revealed improved function and overall satisfaction with the procedure. Subsequent radiotherapy was administered 6 weeks after surgery. Follow-up evaluations indicated a significant reduction in self-reported pain, with continued improvement at the 3-month mark (Table 1). Minimal opioid use was reported after 12 months, demonstrating satisfactory results with a NASS score of 2. Radiographic assessments at each follow-up up to the 12 months visit showed no signs of implant failure or loosening.

### Case 3, 59 years, male, Non-Hodgkin lymphoma stage IVB (Ann- Arbor)

**Fig. 3:**
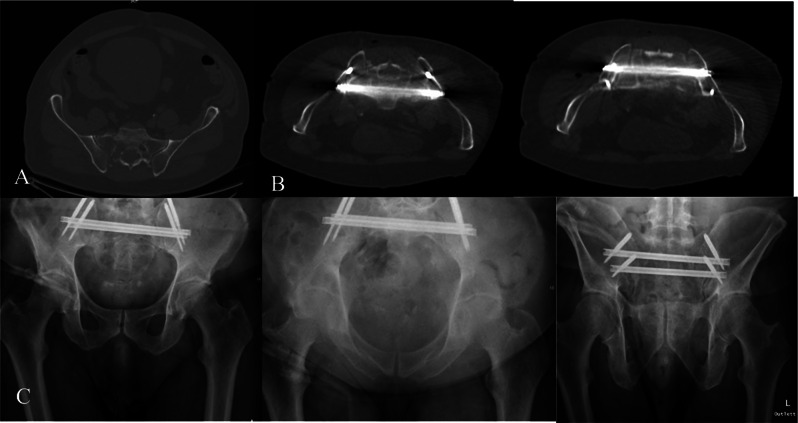
Patient 3 **A**- preoperative CT scan showing sacral fracture, **B**- intraoperative CT scan, **C**- X- Rays demonstrating no implant failure 4 months postoperative

A 59-year-old male with Non-Hodgkin lymphoma in stage IVB (Ann Arbor) had previously undergone one course of chemotherapy (R-Pola-CHP). During the second chemotherapy cycle, he developed immobility and an inability to walk due to significant pelvic involvement (Fig. 3). Four additional chemotherapy courses were planned. The patient struggled to carry out daily activities independently. Surgical intervention provided significant pain relief. The procedure utilized CT navigation (O-arm, Medtronic, Dublin, Ireland), resulting in high patient satisfaction and improved pain management, reflected in a NASS score of 1 (indicating met treatment expectations). The patient could not bear full weight due to overall exhaustion and lumbar plexus invasion. Neurological rehabilitation was initiated. Additionally, a pathological humeral shaft fracture was addressed through osteosynthesis with humeral nailing following the SACRONAIL^®^ placement. No surgical complications were encountered. Chemotherapy resumed 2 weeks post-surgery. Unfortunately, the patient was lost to structured follow-up. During the last consultation 4 months post-surgery, the patient demonstrated full weight-bearing using a walker.

## Discussion and conclusions

The cases presented highlight the benefits of utilizing minimally invasive SACRONAIL^®^ implants for pathological fractures in solid tumor metastases and haematological malignancies, such as lymphomas. The stability provided by the SACRONAIL^®^ allows for immediate full weight-bearing, irrespective of the patient’s treatment timeline for chemotherapy or radiation therapy. All cases showed a significant reduction in pain levels and opioid use after surgery, with high patient satisfaction and a willingness to undergo the procedure again if needed. In this regard, the results are consistent with earlier literature on percutaneous screw treatment, both with and without cement augmentation, and AORIF, which report improvements in pain symptoms and mobility [20, 22, 23]. In our view, the advantage of the SACRONAIL^®^ lies in its very rigid, bilaterally fixated construct in the ilium, which may result in fewer implant failures during the extended fracture healing times expected in pathological fractures. This also allows us to permit early full weight-bearing as tolerated, in contrast to screw fixations. For osteosyntheses in long tubular bones, implant failure rates of up to 22% have been reported after one year [8]. A significantly longer observation period would be needed to capture these potential effects.

The SACRONAIL^®^ demonstrates promising results in terms of stability, early mobilization with full weight-bearing, and pain reduction, which have already influenced the standard treatment of fragility fractures of the pelvis at our institution. However, the small sample size for pathological fractures necessitates further studies with larger case series to validate the positive outcomes observed with this implant system.

## Data Availability

Data generated or analysed during this study are included in this published article. Raw data of the study are not publicly available as the study participants have not given a signed consent for public insight and use and their privacy is respected under the European General Data Protection Regulation. Anonymized raw data are available on request.

## Abbreviations

AORIF: Ablation, Osteoplasty, Reinforcement and Internal Fixation
FFP: Fragility Fracture of the pelvis
MBD: Metastatic Bone Disease
MSS: Mirels Scoring System
NASS: North American Spine Society
NRS: Numeric Rating Scale
